# Creatine Kinase and Respiratory Decline in Amyotrophic Lateral Sclerosis

**DOI:** 10.3390/brainsci14070661

**Published:** 2024-06-28

**Authors:** João Pedro Correia, Marta Gromicho, Ana Catarina Pronto-Laborinho, Miguel Oliveira Santos, Mamede de Carvalho

**Affiliations:** 1 Faculdade de Medicina, Instituto de Medicina Molecular, Universidade de Lisboa, 1649-004 Lisboa, Portugal; joao.pedro.correia@campus.ul.pt (J.P.C.); martalgms@gmail.com (M.G.); anapronto@medicina.ulisboa.pt (A.C.P.-L.); migueloliveirasantos@hotmail.com (M.O.S.); 2 Centro de Estudos Egas Moniz, Faculdade de Medicina, Universidade de Lisboa, 1649-004 Lisboa, Portugal; 3 Departamento de Neurociências e Saúde Mental, Hospital (ULS) de Santa Maria, 1649-028 Lisboa, Portugal

**Keywords:** amyotrophic lateral sclerosis, creatine kinase, non-invasive ventilation, respiratory function, survival

## Abstract

Respiratory dysfunction is an important hallmark of amyotrophic lateral sclerosis (ALS). Elevation of creatine kinase (CK) has been reported in 23–75% of ALS patients, but the underlying mechanisms remain unknown. This work aims to enlighten the role of CK as a prognostic factor of respiratory dysfunction in ALS. A retrospective analysis of demographic and clinical variables, CK, functional decline per month (ΔFS), forced vital capacity (%FVC), and mean amplitude of the phrenic nerve compound motor action potential (pCMAP) in 319 ALS patients was conducted. These measurements were evaluated at study entry, and patients were followed from the moment of first observation until death or last follow-up visit. High CK values were defined as above the 90th percentile (CK ≥ P90) adjusted to sex. We analyzed survival and time to non-invasive ventilation (NIV) as proxies for respiratory impairment. Linear regression analysis revealed that high CK was associated with male sex (*p* < 0.001), spinal onset (*p* = 0.018), and FVC ≥ 80% (*p* = 0.038). CK was 23.4% higher in spinal-onset ALS patients (*p* < 0.001). High CK levels were not linked with an increased risk of death (*p* = 0.334) in Cox multivariate regression analysis. CK ≥ P90 (HR = 1.001, *p* = 0.038), shorter disease duration (HR = 0.937, *p* < 0.001), lower pCMAP (HR = 0.082, *p* < 0.001), and higher ΔFS (HR = 1.968, *p* < 0.001) were risk factors for respiratory failure. The association between high CK levels and poorer respiratory outcomes could derive from cellular metabolic stress or a specific phenotype associated with faster respiratory decline. Our study suggests that CK measurement at diagnosis should be more extensively investigated as a possible marker of poor respiratory outcome in future studies, including a larger population of patients.

## 1. Introduction

Creatine kinase (CK) and the creatine/phosphocreatine (Cr/PCr) system are key players in normal cellular function, especially in high-energy demand tissues, such as skeletal muscle and brain, sites that are invariably implicated in amyotrophic lateral sclerosis (ALS). For over 50 years, abnormal elevation of serum CK values has been reported in up to 75% of ALS patients [1,2], and early studies have attributed this increase as a consequence of muscle cell death driven by progressive motor neuron degeneration [3]. An alternative theory, considering the role of CK in cellular bioenergetics, considers it may be upregulated in the face of metabolic stress [4]. In fact, it remains elusive if CK elevation in ALS patients is due to a neurogenic process or a metabolically mediated primary myopathic phenomenon.

**Figure 1:**
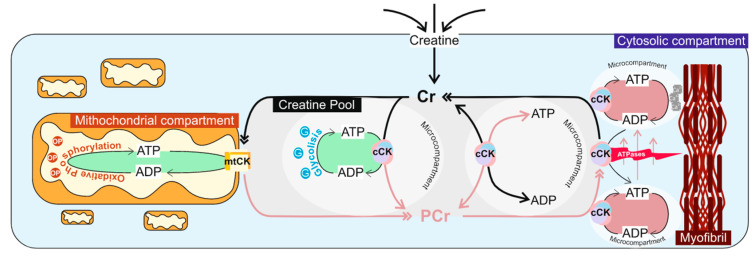
Schematic representation of the creatine kinase/phosphocreatine (CK/PCr) system illustrating the ability of the CK/PCr system to function as a spatial and temporal energy buffer. As a spatial buffer, the CK/PCr system transports phosphoryl compounds from mitochondria or glycolytic enzymes to different microcompartments within the cell, where ATP is utilized (excitation-contraction coupling: myosin ATPase, in the myofibrils, and Ca^2+^-ATPase, in the sarcoplasmic reticulum) as a temporal buffer, the energy reservoir produced by the CK/PCr system can buffer pH (by utilizing H^+^) and ADP accumulation to help to maintain more favorable energetic conditions during periods of high metabolic demand. Legend: ATP—adenosine triphosphate; ADP—adenosine diphosphate; Cr—creatine; PCr—phosphocreatine; mtCK—mitochondrial CK; cCK—cytosolic CK.

PCr is the primary source of adenosine triphosphate (ATP) supply in mammal skeletal muscles during the transition from rest to activity [5], whereby, to keep ATP constant, PCr is rapidly consumed, as a short-term energy buffer, in which adenosine diphosphate (ADP) is phosphorylated to ATP, a reaction that is catalyzed by CK [6]. Inside cells, there are also multiple mitochondrial and cytoplasmic compartments interconnected by a coordinated energy transfer system constituted by different isoforms of CK [7]. These particularities attribute CK an important role in the mediation of cellular homeostasis since it can reversibly convert Cr into PCr to create a large pool of PCr for temporal and spatial ATP buffering [8] (Figure 1).

To further comprehend the use of CK as a biomarker in ALS, the current literature was reviewed (Table A1). There are several factors that can explain the conflicting data on the role of CK in ALS patients, including methodological differences between studies, different reference ranges for serum CK, and a lack of consensus on what constitutes a high CK level. Altogether, the literature review suggests that muscle mass is the main determinant of the degree of CK rise, although it remains elusive what is the underlying mechanism(s) that lead to the elevation of serum CK in ALS patients.

Previous reports have shown that male sex, spinal onset [1,2], muscle cramps [2], higher functional status [9], predominant lower motor neuron involvement [1], and recent loss of motor units on EMG [10] are significantly associated with high CK, but no association with fasciculations was disclosed [2,11]. These findings are in favor of a neurogenic origin of the increased CK levels. In general, high CK was associated with longer survival [1], but the opposite finding has been described [2]. Interestingly, longitudinal CK measurements show stable values [1,11]. The main cause of death in ALS is respiratory complications; therefore, our aim is to investigate the relationship between CK and respiratory failure, defined as the time to non-invasive ventilation (NIV), in ALS.

## 2. Patients and Methods

This retrospective study analyzed a cohort of ALS patients diagnosed between 1998 and 2022 at a main tertiary hospital in Lisbon (Portugal). Information was collected from a database with demographic and clinical data. At the time of the first observation (baseline), relevant data were registered using a standardized questionnaire.

### 2.1. Study Participants and Clinical Parameters

All patients were first observed by the same neurologist with extensive experience in ALS (MdC). In almost all patients, riluzole was initiated following the first observation of ALS in our center; in a few cases, it was started before.

We included patients with progressive muscular atrophy and categorized them as possible, probable, and definite diseases on El Escorial Criteria [12]. Patients with lung diseases, heart failure, or other neurological conditions were excluded. Patients with severe respiratory symptoms, absent phrenic nerve responses, or unable to perform respiratory tests were not included in this study. Considering the aim of this study, patients were considered eligible if a single serum CK measurement was available within 100 days of study entry (first visit to our center).

Demographic and anthropometrical variables, as well as respiratory function data, were also sourced from the database. Patients were asked if they had fasciculations at disease onset, after a brief explanation of the signs and symptoms associated. In cases where data were missing, medical records were reviewed to ensure the completeness of each participant’s data set.

In all patients, needle electromyography (nEMG) was performed in the upper (UL) and lower (LL) limbs as part of the diagnostic workup. The functional impairment was assessed by the ALSFRS-R. The disease progression rate was calculated by the change rate of ALSFRS-R (ΔFS), using the formula: 48—ALSFRS-R score at the baseline visit/disease duration in months. Fast and average progressors were those with scores that declined over 0.5 points per month (ΔFS ≥ 0.5), while slow progressors lost less than 0.5 points per month (ΔFS < 0.5) [13,14].

To account for the variability in CK assay measurements, the cut-off for high CK was defined as above the 90th percentile (P90) for men and woman ALS patients, considering the normative values at the time of measurement.

Height and weight were measured following international guidelines, and the body mass index (BMI) was calculated as weight in kilograms divided by the square of height in meters. In the absence of direct methods to quantify body composition indices, fat body mass (eFBM) was estimated from BMI using the formula proposed by Gallagher and colleagues [15] and expressed as a percentage. Fat-free mass (eFFM) was estimated using the equation Weight × [1 − (FBM%/100)] and expressed in kilograms (kg). The resting energy expenditure (cREE) was calculated as a surrogate measure of the basal metabolic rate using the formula proposed by Jésus and colleagues for ALS patients [16].

### 2.2. Measures of Respiratory Function

The parameters used to assess respiratory status at ALS first observation included the ALSFRS-R respiratory sub-scores, percentage of the predicted value for forced vital capacity [17], and mean peak-to-peak amplitude value of both left and right diaphragm compound muscle action potentials (CMAP) elicited by phrenic nerve stimulation (pCMAP). FVC and pCMAP were obtained within a period of 100 days from study entry.

FVC was measured in the sitting position using an adequately calibrated hand-held portable spirometer. The best of three acceptable and repeatable FVC measurements was used for statistical analysis and expressed as a percentage of the predicted value (%FVC). Patients were allocated to two groups, those with normal (≥80%) and decreased (<80%) FVC.

pCMAP was derived from percutaneous bipolar electrical phrenic nerve stimulation at the neck with the active electrode positioned at the ipsilateral costosternal angle and the reference electrode at the costal margin 16 cm from the active electrode [18]. The responses were measured at the end of expiration until three consistent motor responses were recorded on either side of the diaphragm. For statistical analysis, the mean bilateral phrenic CMAP nerve amplitude (L+R/2) motor response was used and expressed in millivolts (mV). Patients were grouped into those with normal (≥0.4 mV) and abnormal (<0.4 mV) responses [19].

The ALSFRS-R respiratory sub-scores were used to determine if there was a respiratory compromise at first evaluation. Patients were grouped into those with no respiratory involvement (12) and those with any degree of respiratory compromise (<12).

### 2.3. Respiratory Decline and Survival Analysis

Patients were followed from the moment of first observation until death or last follow-up visit. The observed endpoint was the event onset of NIV (Time to NIV) or time of death (survival time). Time to NIV was used as a proxy of respiratory failure and defined as the difference between the date of symptom onset and the date when NIV was recommended. NIV introduction was established by an independent ventilatory support team, according to the European guidelines [20]. Survival and NIV status was last updated on 31 December 2022.

Multivariate Cox analyses were used to examine the effect of serum CK values on survival and respiratory failure adjusting for covariates known to have an effect on prognosis, such as age, onset site, disease duration, BMI, %FVC, pCMAP, and ΔFS.

### 2.4. Statistical Analysis

Continuous variables were checked for normal distribution using qq-plots, skewness, and kurtosis statistics and described with their mean and standard deviation (SD). Group comparisons for categorical data were conducted using χ^2^ or Fisher’s exact test, depending on the number of cases. Group comparisons for ordinal and metric data were performed using the student’s *t*-test or Mann–Whitney U test, according to the distribution of the data. The correlation between serum CK values and continuous variables was assessed using the Spearman coefficient (*r_s_*) with a graphical representation of the relationships using scatter plots. Correlations with a *p*-value <0.01 were considered statistically significant.

Separate linear regression models were implemented using serum CK, FVC, pCMAP, and (ΔFS) as the dependent variables. To ensure linear regressions assumptions were met (linearity, independence of observations, residuals homoscedasticity, and residuals normality), there was a need to perform a logarithmic transformation to CK (lg10 CK) and pCMAP (lg10 Phre) and square root transformation to FVC (sqrtFVC) and an inverse transformation to the function decline (1/ΔFS). First, a univariate regression analysis was performed for the variables: age at diagnosis, disease duration, BMI, eFFM, eFBM, FVC, and ΔFS. Variables associated with a risk of multicollinearity and with a *p*-value ≥0.20 in the univariate analysis were excluded from the multivariate model.

Survival analysis was performed by univariate Kaplan–Meier log-rank and multivariate Cox proportional hazards. All *p*-values were two-sided, and values <0.05 were deemed statistically significant. Statistical analysis was performed using SPSS^®^ 28.0 software (IBM Corp., Chicago, IL, USA, 2021).

## 3. Results

Of the initial cohort (*n* = 755), 319 patients (42.3%) for whom serum CK measurement was available were included in the analysis censored, and missing data are detailed in Table 1, Table 2 and Table 3. The mean age of disease onset was 61.4 ± 13.0 (mean ± SD) years, with a mean disease duration of 16.7 ± 17.9 months at the time of first observation in our unit. Men were more frequent (59.1%). Up to 75% of cases were spinal-onset ALS, with fasciculations reported in 36.8% of cases. Needle EMG revealed abnormal findings in the upper (UL) and lower (LL) limbs in 86.3% of cases, whilst isolated UL or LL involvement in the remainder (13.7%) (Table A2). The mean functional decline rate was 0.85 ± 1.05, and a rate ≥ 0.5 (average/fast progressors) was observed in 55.6% of patients. The median time between disease-onset and the start of NIV (Time to NIV) was 34.00 months (1st and 3rd IQR: 26.96–41.05), whereas the median survival time was 50.27 months (1st and 3rd IQR: 42.70–57.83). There was a statistically significant difference in the age of disease onset by sex (*p* = 0.004), with women being older. Men had a higher prevalence of spinal-onset, whereas females showed bulbar-onset sites to be more prevalent (*p* = 0.002). There were no statistically significant differences between sexes regarding other variables (nEMG UL/LL involvement and disease duration).

**Figure 2:**
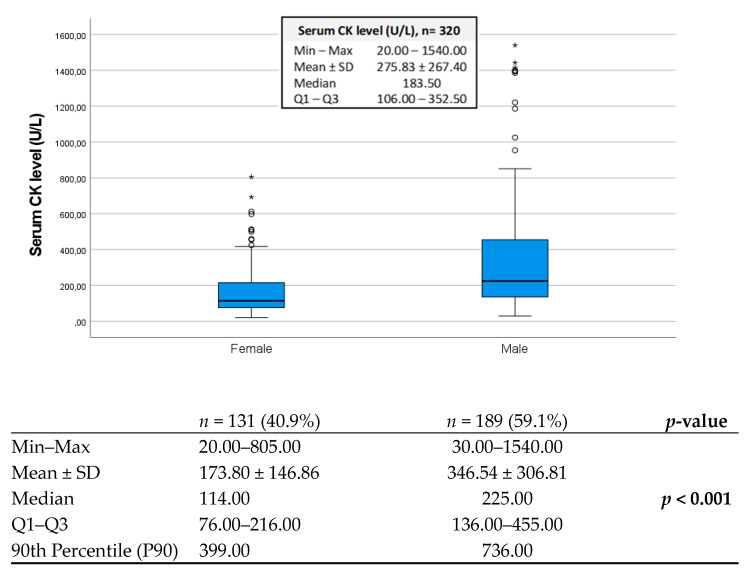
Boxplot diagram of serum CK values in ALS participants: men and women. Results presented Min–Max, mean ± SD, medians, quartiles (Q1–Q3), and the 90th percentile (P90) for serum CK; significance was calculated with the Mann–Whitney test. Bold values show *p*-values < 0.05. **Legend: CK**—creatine kinase; **ALS**—amyotrophic lateral sclerosis.

Since there were technical changes in CK determination over the period of recruitment, in spite of no considerable differences concerning upper reference ranges, it was not deemed appropriate to determine the prevalence or the diagnostic value of increased serum CK in this cohort. There were 9 patients (2.8%) with serum CK values ≥ 1000 U/L, all of which were men. Serum CK values were significantly higher in men (*p* < 0.001), with a median value of 225 U/L (1st and 3rd IQR: 136–455 U/L), when compared with women, with a median value of 114 U/L (1st and 3rd IQR: 76–216 U/L), as illustrated in Figure 2.

Given the differences between sexes, further comparisons with CK and the other variables in the study were performed separately for sex using the 90th percentile (P90) as the criteria for dichotomization (low/normal CK < P90 vs. high CK ≥ P90). The P90 CK was 736 U/L and 399 U/L, for men and women, respectively.

In men, CK was significantly higher in patients with an age below 65 years (*p* < 0.001), spinal-onset (*p* = 0.001), and patients with both UL and LL involvement objectified by nEMG (*p* = 0.006) (Table A3). In women, the only significant difference observed was between onset sites, with higher CK in spinal-onset (*p* < 0.001) (Table A4). No statistically significant association was noted with fasciculations at presentation nor with functional rate of decay in both sexes. In men, CK ≥ P90 was associated with a younger age at diagnosis (*p* = 0.013) (Table A5). In women, the cohort with CK values ≥ P90 was represented only by spinal-onset patients, and no variable was found associated with this subgroup (Table A6).

We tested the studied variables influencing CK value (dependent variable) using a regression model (Table 1). We applied a logarithmic transformation of CK values (lg10 CK). The univariate analysis revealed a significant negative association with age at diagnosis (β = −0.006, *p* < 0.001) and eFBM (β = −0.016, *p* < 0.001) and a significant positive association with eFFM (β = 0.014, *p* < 0.001). Bulbar-onset patients were associated with lower CK values (β = −0.274, *p* < 0.001), whilst men were associated with higher CK (β = 0.286, *p* < 0.001). In the multivariate model, including variables with *p*-value > 0.2, the only significant predictor of lg10 CK higher values was the onset site (β = −0.234, *p* < 0.001). Bulbar-onset patients were predicted to have 23.4% lower CK values than limb-onset patients.

### CK and Respiratory Function

The respiratory function variables were compared between the cohort with CK ≥ P90 and CK < P90 for the overall study population (Table A7). ALS patients with CK values ≥ P90 had higher %FVC (*p* = 0.038), with no significant differences noted for the ALSFRS-R respiratory sub-scores and pCMAP. Spearman-ranked correlation between CK and respiratory function variables found non-significant results for %FVC (*p* = 0.216) and pCMAP (*p* = 0.261).

**Figure A1:**
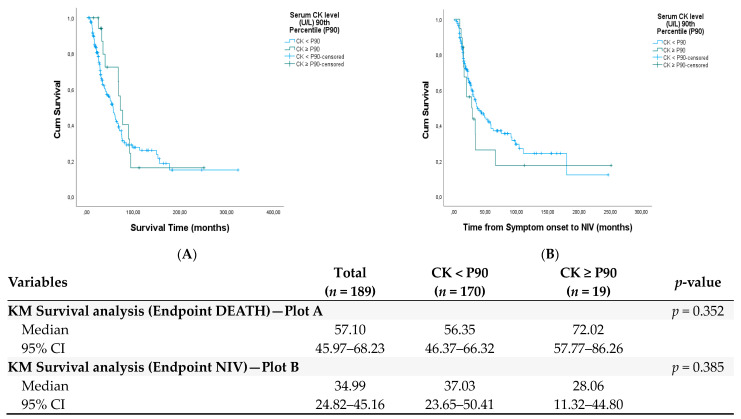
Kaplan–Meier (KM) survival analysis for endpoint death (**A**) and NIV (**B**) in men population in association with 90th percentile (P90) CK (736 U/L). Time-to-event graphs comparing male ALS patients’ survival with serum CK < 90th percentile and ≥ 90th percentile. (**A**) Kaplan–Meier analysis to time-to-death and (**B**) Kaplan–Meier analysis to time-to-NIV. Results were presented as Min–Max, medians, and quartiles (Q1–Q3); significance was calculated with the log-rank test. Bold values show *p*-values <0.05. **Legend: NIV**—non-invasive ventilation; **CK**—creatine kinase; **KM**—Kaplan–Meier.

**Figure A2:**
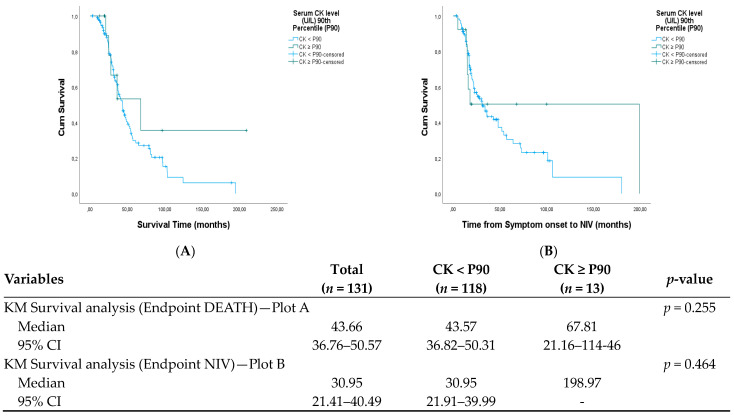
Kaplan–Meier (KM) survival analysis for endpoint death (**A**) and NIV (**B**) in the female population in association with 90th percentile (P90) CK (399 U/L). Time-to-event graphs comparing female ALS patients’ survival with serum CK < 90th percentile and ≥ 90th percentile. (**A**) Kaplan–Meier analysis to time-to-death and (**B**) Kaplan–Meier analysis to time-to-NIV. Results were presented as Min–Max, medians, and quartiles (Q1–Q3); significance is calculated with the log-rank test. **Legend: NIV**—non-invasive ventilation; **CK**—creatine kinase; **KM**—Kaplan–Meier.

To explore if high serum CK values had any impact on respiratory failure (endpoint: NIV) and survival (endpoint: death), a Kaplan–Meier analysis was performed by comparing the cohort with CK ≥ P90 and CK < P90. In men, there was no statistically significant difference between the cohorts in survival (*p* = 0.352) and time to NIV (*p* = 0.385) (Figure A1). An identical observation was noted in females when comparing the cohorts with CK ≥ P90 and CK < P90 in survival (*p* = 0.255) and time to NIV (*p* = 0.464) (Figure A2).

To determine the influence of the different variables in a study on respiratory failure and survival time, a Cox proportional hazards model was computed including the whole population. An increased risk of death was associated with older age at ALS diagnosis (HR = 1.031, *p* < 0.001), bulbar-onset site (HR = 1.760, *p* = 0.001), shorter disease duration (HR = 0.979, *p* < 0.001), lower BMI (HR = 0.952, *p* = 0.016), and higher ALSFRS-R functional decline (HR = 2.485, *p* < 0.001) (Table 2).

For the endpoint NIV, the multivariate Cox regression analysis revealed that a shorter disease duration (HR = 0.937, *p* < 0.001), higher CK values (HR = 1.001, *p* = 0.038), lower pCMAP (HR = 0.082, *p* < 0.001), and a higher ALSFRS-R functional decline (HR = 1.968, *p* < 0.001) were associated with an increased risk of commencing NIV therapy (Table 3).

## 4. Discussion

In general, CK is elevated in about 40–50% of ALS patients (Table A1) but is a non-specific finding as it can be elevated in other similar neuromuscular conditions, such as spinal and bulbar muscular atrophy.

Our results found that spinal onset is the main determinant of high CK values, which tend to be higher in athletic (higher eFFM) young men. The very first descriptions that documented raised serum CK values in ALS patients have systematically attributed this to a leakage of the enzyme across cellular membranes in damaged or denervated muscle tissue, with the degree of CK rise affected by the rate of the atrophic process and the volume of muscle mass involved at the time of measurement [21]. According to Chahin and Sorenson [22], ALS patients with longer disease duration have a higher prevalence of myopathic changes in muscle fibers; justifying this way, how some ALS patients have raised CK values, and others have not. The relevance of myopathic abnormalities has been extensively discussed recently [23]. Ito and colleagues [24] suggested that raised serum CK levels likely reflect denervation during the pre-clinical stage of ALS due to membrane instability, hyperexcitability of motor neurons, and destruction of denervated muscle tissue, and the degree of elevation could reflect the proportion of type I and type II muscle fibers affected. Rafiq [4] adjusted serum CK to estimated lean body mass (eLBM) and noted that high serum CK levels were still significantly associated with longer survival, hence suggesting that the prognostic benefit of upregulation in CK expression is not uniquely dependent on muscle mass and could be attributed to the ability to provide energy for the hypercatabolic nature of ALS. Some authors assume that a higher volume of skeletal muscle mass translates to a higher muscular metabolic reservoir; this is the reason why ALS patients with a slow progression rate have higher serum CK values [25]. These descriptions reiterate that, in ALS, the pathogenesis of serum CK elevation remains unknown.

Based on the literature review (Table A1), one single study evaluates CK values in respiratory-onset patients showing significantly lower values when compared to limb and bulbar-onset patients [26], probably due to the important muscle atrophy and weight loss typical of this phenotype [27]. Respiratory muscles correspond to a small proportion of the whole-body bulk of skeletal muscle mass; thus, their involvement should not result in a significant elevation of CK values. Even so, there were no studies evaluating if serum CK values have an influence on respiratory function and on the rate of respiratory decline.

We have observed that high CK values were not associated with poorer performance on the respiratory tests at baseline. In addition, high CK values were not a prognostic factor for survival. However, Cox regression analysis demonstrated that high CK values were predictive of the need for NIV, besides shorter disease duration, lower pCMAP, and steeper functional decline. Considering that CK is a marker of muscular necrosis, higher CK values could imply a faster loss of skeletal muscle mass with earlier involvement of the respiratory muscles. Moreover, it could be that higher CK is part of a spinal-onset phenotype associated with faster disease progression.

Our study has several limitations, in particular, patients were included at first observation in our center, which in some patients it was a few months after diagnosis in another center. Other methods to evaluate diaphragm movement, like ultrasound, were not applied. Genetic tests were only available for a small subgroup of patients, precluding to investigation of the role of different mutations on the CK level. Laboratory techniques to determine CK values have changed over the years. There is no perfect proxy for respiratory dysfunction in ALS, so we decided on the time to NIV based on independent specialists following the European Guidelines; however, time to NIV depends on personal decisions that cannot be quantified. Furthermore, regarding the predictive value of high CK to determine NIV indication, the lower bound 95% confidence interval of the hazard ratio equals 1.0, meaning that there is a possibility that no added risk is associated with higher CK values.

Our study suggests that CK measurement at diagnosis is a simple test giving relevant clinical information, since a very high value indicates a greater chance of poor respiratory outcome, recommending that these patients should be monitored more frequently.

It remains to answer the question about the possible meaning of hyperCKemia in the respiratory outcome of ALS patients. The role of CK in molecular bioenergetics and its plasticity in supporting the cell energy requirements appear to be beneficial during early disease stages; but as the disease progresses, this metabolic upregulation has a cost, boosted by the shift from glycolytic to β-oxidation as the main energy substrate, consuming more oxygen to produce ATP and consequently increasing reactive oxygen species and toxic metabolites. Moreover, this higher β-oxidation use can affect mitochondrial respiratory capacity. It may be that all patients diagnosed with ALS share a common natural history in serum CK values that, although variable, may well be representative of distinct disease stages. This raises the question of whether the true prognostic value of CK does not rely on a one-off determination or serial longitudinal assays during disease progression, but on the ability and degree of variability in CK rise in the face of metabolic stress. Further studies are necessary.

## Figures and Tables

**Table 1:** Linear regression model between CK logarithmic transformation (lg10 CK), demographics, disease characteristics, and anthropometrical variables.

Variables	Univariate Analysis			Multivariate Analysis *		
	*n*	*p*-Value	*β* 95% CI	*n*	*p*-Value	*β* 95% CI
Sex	319	**<0.001**	(0.286; 0.208, 0.363)	172	0.256	(0.189; −0.138, 0.516)
Age at diagnosis	319	**<0.001**	(−0.006; −0.010, −0.003)	“	0.056	(−0.005; −0.011, 0.000)
Site of onset	319	**<0.001**	(−0.274; −0.363, −0.184)	“	**<0.001**	(−0.234; −0.349, −0.118)
Disease duration (months)	319	0.166	(0.002; −0.001, 0.004)	“	0.497	(0.001; −0.002, 0.004)
Body mass index (kg/m^2^)	270	0.370	(0.005; −0.007, 0.018)		(x)	
Fat-free mass (kg)	270	**<0.001**	(0.014; 0.009, 0.018)	“	0.824	(0.001; −0.011, 0.014)
Fat body mass (%)	270	**<0.001**	(−0.016; −0.021, −0.010)	“	0.890	(−0.001; −0.014, 0.012)
Forced vital capacity (%pred)	180	0.198	(0.002; −0.001; 0.004)	“	0.902	(0.000; −0.002, 0.002)
Mean phrenic nerve CMAP amplitude (mV)	208	0.313	(0.092; −0.087, 0.272)		(x)	
ALSFRS-R functional decline (ΔFS)	319	0.070	(−0.036; −0.075, 0.003)	“	0.985	(0.000; −0.048, 0.047)

* Independence of residuals was confirmed by a Durbin–Watson statistic of 1.769; partial regression plots revealed homoscedasticity and a linear relationship between lg10 CK and the independent variables; outliers were not removed from data analysis; variables with *p*-value > 0.20 in univariate analysis (x) were excluded from multivariate analysis [F(8, 164) = 7.949, *p* < 0.001]. The coefficient of determination (R^2^) for the overall model was 27.9% with an adjusted R^2^ of 24.4%, a medium-size effect according to Cohen (1988). Bold values show *p*-value < 0.05. **Legend: CK**—creatine kinase.

**Table 2:** Cox multivariate regression analysis for the time from diagnosis until death/censorship including the whole population.

Variables	Cox Multivariate Analysis—Endpoint Death			
	*n* *	*p*-Value	HR	95% CI
Age at diagnosis	269	**<0.001**	1.031	(1.017, 1.045)
Onset site (reference-bulbar)	“	**0.001**	1.760	(1.242, 2.494)
Disease duration (months)	“	**<0.001**	0.979	(0.967, 0.991)
Serum CK level (U/L)	“	0.334	1.000	(1.000, 1.001)
Body mass index (kg/m^2^)	“	**0.016**	0.952	(0.915, 0.991)
ALSFRS-R functional decline (ΔFS)	“	**<0.001**	2.485	(2.041, 3.027)

* 49 cases with missing values and 2 censored cases before the earliest event in the stratum. Bold values show *p*-values <0.05. **Legend: CK**—creatine kinase.

**Table 3:** Cox multivariate regression analysis for the time from diagnosis until the start of non-invasive ventilation/censorship including the whole population.

Variables	Cox Multivariate Analysis—Endpoint NIV			
	*n* *	*p*-Value	HR	95% CI
Age at diagnosis	155	0.507	0.994	(0.977, 1.012)
Onset site (reference-bulbar)	“	0.247	1.349	(0.812, 2.241)
Disease duration (months)	“	**<0.001**	0.937	(0.910, 0.964)
Serum CK level (U/L)	“	**0.038**	1.001	(1.000, 1.002)
Body mass index (kg/m^2^)	“	0.509	1.019	(0.963, 1.078)
Forced vital capacity (%pred)	“	0.384	0.995	(0.984, 1.006)
Mean phrenic nerve CMAP amplitude (mV)	“	**<0.001**	0.082	(0.025, 0.273)
ALSFRS-R functional decline (ΔFS)	“	**<0.001**	1.968	(1.357, 2.854)

* 164 cases with missing values. Bold values show *p*-values <0.05. **Legend: CK**—creatine kinase.

## Data Availability

The data presented in this study are available on request from the corresponding author. The data are not publicly available due to privacy and ethical reasons.

## Appendix B

**Table A1:** Relevant publications * evaluating CK in ALS patients.

Author/Publication Date (Country)	Methods and Study Population	Observations
Harrington et al., 1983 (USA) [3]	Retrospective study; 100 ALS patients with CK during clinical evaluation.	43% of patients had increased CK; Spinal onset and muscle cramps >> CK.
Sinaki and Mulder, 1986 (USA) [28]	Retrospective study; 30 ALS patients with CK at diagnosis.	50% of patients had increased CK; No association with survival time and muscular weakness.
Felice and North, 1998 (USA) [29]	Retrospective study; 140 ALS patients with CK at diagnosis; 48 repeated the analysis.	41% of patients had increased CK; Males and spinal onset >> CK; Weak negative correlation with age and survival time.
Lima et al., 2003 (Portugal) [11]	Retrospective study; 87 ALS patients with CK and 22 repeated the analysis.	43% of patients had increased CK values (?); No significant differences in sex and site of onset; No association with fasciculations and signs of denervation.
Süssmuth et al., 2003 (Germany) [30]	Prospective study; 20 ALS patients with CK and 7 patients repeated the analysis.	70% of patients had increased CK values; CK increased during the first 18 months after disease onset.
Iłzecka and Stelmasiak, 2003 (Poland) [31]	Retrospective study; 30 ALS patients with CK during clinical evaluation.	43.3% of patients had increased CK levels (?); Spinal onset >> CK; no significant difference in sex; No association with age, disease duration, and ALS severity.
Chahin and Sorenson, 2009 (USA) [22]	Retrospective study; 36 ALS patients with CK during clinical evaluation.	23% of patients had increased CK values; No association with ALSFRS and age.
Chiò et al., 2014 (Italy) [32]	Retrospective study; 638 ALS patients with CK during clinical evaluation.	Males >> CK; No influence on survival.
Gibson et al., 2015 (USA) [2]	Prospective study; 68 ALS patients with serial CK at baseline, 16, 32, and 48 weeks.	Males, spinal onset, and muscle cramps >> CK; Decreasing CK kinetics over the 48-week study period; Significant hazard of death >> CK.
Rafiq et al., 2016 (Europe) [4]	Prospective study; 512 ALS patients with CK over a period of 18 months (at 3-month intervals).	52% of patients had increased baseline CK values; Males, spinal onset, lower limb involvement >> CK; Decreasing CK kinetics over the 18-month study period; Strong positive association with eLBM; Significant survival benefit >> CK.
Lu et al., 2016 (United Kingdom) [33]	Prospective study; 95 ALS patients with CK over a period of 48 months (2- to 4-month intervals).	High ALSFRS-R score >> CK; High disease progression rate << CK; Positive association with IL-5.
Tai et al., 2017 (China) [34]	Retrospective study; 185 ALS patients with CK during clinical evaluation.	43% of patients had increased CK values; Males and spinal onset >> CK; Significant association with sex, creatinine, and site of onset EMG: Significant correlation with spontaneous potentials (scaled 1–10 points as per location, intensity of fibrillation potential, positive sharp wave, and fasciculation potentials); No association with age, BMI, and disease duration; Significant survival benefit >> CK.
Ong et al., 2017 (Worldwide) [35]	Retrospective study of ALS patients’ data within 3 months of diagnosis from the PRO-ACT database.	Slow progressors >> CK; Fast progressors had a more pronounced decrease in CK.
Tai et al., 2018 (China) [10]	Retrospective study; 238 ALS patients with CK at ALS diagnosis.	38% of patients had increased CK values; Males and spinal onset >> CK; EMG: Significant association in males with high denervation potential (F-wave persistence in the median nerve).
Wei et al., 2018 (China) [36]	Prospective study; 553 ALS patients with CK at diagnosis and followed up every 3 to 6 months.	Significant survival benefit >> CK
Ito et al., 2019 (Japan) [24]	Prospective study; 39 ALS patients with CK at ALS diagnosis; CK was retrospectively analyzed in 8 patients.	CK was elevated prior to onset, reaching peak values at onset, with decreasing kinetics from onset onwards; Weak association with ALSFRS-R; No association with a decline in motor function.
Ceccanti et al., 2020 (Italy) [25]	Prospective study; 126 ALS patients with serial CK over a period of 16 months (every 4 months).	47% of patients had increased CK values; Spinal onset and slow progressors >> CK; Fast progressors had stable CK values over time.
Guo et al., 2021 (China) [37]	Prospective study; 346 ALS patients with CK over a period of 12 months (3- to 6-month intervals).	Males >> CK; Significant association with body weight; No influence on survival.
Chen et al., 2021 (China) [38]	Retrospective study; 582 ALS patients with CK and 81 patients repeated the analysis (>6 months).	Males and spinal onset >> CK; Significant positive association with creatinine, UA, total protein, albumin, LDL-C, total cholesterol; No association with ALSFRS-R score; Significant survival benefit >> CK.
Chang et al., 2022 (Republic of Korea) [26]	Retrospective study with laboratory results obtained at diagnosis of 115 ALS patients with respiratory, bulbar, and limb onset.	CK was higher in patients with limb onset (250 ± 228.2), followed by those patients with bulbar (137.6 ± 79.4) and respiratory onset (63.6 ± 31.3).
Gao et al., 2022 (United Kingdom) [1]	Prospective study; 222 ALS patients with CK and 91 patients repeated the analysis (3- to 6-month intervals).	39% of patients had increased CK values; Males, spinal onset, and LMN burden >> CK; Significant positive association with eLBM; Weak negative association with progression rate and UMN burden; No temporal trend observed over time; No influence on survival.
Hertel et al., 2022 (Europe) [9]	Retrospective study; 817 ALS patients with CK within 1 year of ALS diagnosis.	Males, younger age at diagnosis, LMN burden, and lower limb involvement >> CK; Low ALSFRS-R score associated with low CK at study entry; No influence on survival.

* The articles included in the review were published between 1980 and the date that the literature search was completed (January 2023); **>>**—higher CK values; **<<**—lower CK values; **(?)**—reference range for males and females was not specified; **EMG**—electromyography; **UA**—uric acid; **LDL-C**—low-density lipoprotein cholesterol; **FFMibs**—fat-free mass measured by bioelectric impedance spectroscopy; **BMI**—body mass index; **eLBM**—estimated lean body mass as per Boer formula.

**Table A2:** Demographics and ALS clinical variables of the study subject’s comparisons by sex.

Variables	Total (*n* = 320)	Male *n* = 189 (59.1%)	Female *n* = 131 (40.9%)	*p*-Value
**Age at diagnosis (years)**, n = 320				***p* = 0.004** (a)
Mean ± SD	61.36 ± 12.98	59.62 ± 13.04	63.87 ± 12.53	
Median	62.00	60.00	66.00	
Q1–Q3	52.50–71.50	50.00–71.00	55.00–73.00	
**Onset site** n (%), n = 320				***p* = 0.002** (b)
Spinal	239 (74.7%)	153 (81.0%)	86 (65.6%)	
Bulbar	81 (25.3%)	36 (19.0%)	45 (34.4%)	
**Fasciculations at disease onset** n (%), n = 299	110 (36.8%)	81 (46.0%)	29 (23.6%)	***p* < 0.001** (b)
**Needle EMG** n (%) n = 285				*p* = 0.499 (b)
UL or LL abnormal	39 (13.7%)	25 (14.9%)	14 (12.1%)	
UL and LL abnormal	245 (86.3%)	143 (85.1%)	102 (87.9%)	
**Disease duration** (months), n = 320				*p* = 0.684 (c)
Mean ± SD	16.68 ± 17.87	17.72 ± 20.50	15.19 ± 13.10	
Median	11.55	11.53	11.56	
Q1–Q3	6.88–19.58	6.57–20.01	6.97–19.06	

Results were presented as n (%) for categorical variables and Min–Max, mean ± SD, medians, and quartiles (Q1–Q3) for continuous variables; significance was calculated with (a) *t*-tests, (b) chi-square tests, and (c) Mann–Whitney tests. Bold values show *p*-values < 0.05. **Legend**: **CK**—creatine kinase; **ALS**—amyotrophic lateral sclerosis; **SD**—standard deviation; **UL**—upper limb—; **LL**—lower limb; **EMG**—electromyography.

**Table A3:** Demographics and ALS clinical variables in association with CK in men.

Variables	Serum CK Level (U/L) in Males (*n* = 189)			*p*-Value
	Mean ± SD	Median	Q1–Q3	
**Age at diagnosis (years)**, n = 189				***p* < 0.001**
<65 years	400.30 ± 333.64	292.00	162.00–533.00	
≥65 years	270.04 ± 246.41	190.00	117.00–323.00	
**Onset site**, n = 189				***p* = 0.001**
Spinal	370.84 ± 314.36	256.00	150.00–475.00	
Bulbar	243.25 ± 250.82	160.00	108.00–222.00	
**Fasciculations at onset**, n = 176				*p* = 0.608
No	350.07 ± 319.74	221.00	132.00–458.00	
Yes	350.93 ± 286.24	239.00	148.00–502.00	
**Needle EMG**, n = 168				***p* = 0.006**
UL or LL abnormal	259.48 ± 328.51	162.00	116.00–206.00	
UL and LL abnormal	375.62 ± 312.24	269.00	140.00–522.00	
**Disease duration** (months), n = 189				*p* = 0.643
<12 months	324.57 ± 274.13	221.00	138.50–435.50	
≥12 months	371.22 ± 339.71	239.00	133.00–472.00	
**Functional decline at 1st visit**, n = 189				*p* = 0.160
Slow progressors (ΔFS < 0.5)	395.42 ± 367.97	239.00	149.50–508.00	
Fast/average progressors (ΔFS ≥ 0.5)	296.09 ± 217.90	219.00	129.00–424.00	

Results were presented as mean ± SD, medians, and quartiles (Q1–Q3) for serum CK level in males; significance was calculated with Mann–Whitney tests. Bold values show *p*-values < 0.05. **Legend**: **CK**—creatine kinase; **ALS**—amyotrophic lateral sclerosis; **SD**—standard deviation; **UL**—upper limb; **LL**—lower limb; **EMG**—electromyography.

**Table A4:** Demographics and ALS clinical variables in association with CK in women.

Variables	Serum CK Level (U/L) in Females (*n* = 131)			*p*-Value
	Mean ± SD	Median	Q1–Q3	
**Age at diagnosis (years)**, n = 131				*p* = 0.725
<65 years	174.03 ± 140.59	116.00	79.00–220.00	
≥65 years	173.62 ± 152.78	111.50	71.00–212.50	
**Onset site**, n = 131				***p* < 0.001**
Spinal	209.29 ± 163.63	150.00	94.00–283.00	
Bulbar	105.98 ± 69.54	84.00	59.00–144.00	
**Fasciculations at onset**, n = 123				*p* = 0.314
No	175.79 ± 155.43	111.50	73.00–216.00	
Yes	187.49 ± 132.63	154.00	96.00–242.00	
**Needle EMG**, n = 116				*p* = 0.635
UL or LL abnormal	202.00 ± 160.78	112.50	80.00–387.00	
UL and LL abnormal	175.98 ± 148.91	130.00	72.00–216.00	
**Disease duration** (months), n = 131				*p* = 0.727
<12 months	178.55 ± 153.86	113.00	73.50–215.00	
≥12 months	168.02 ± 138.92	116.00	76.00–220.00	
**Functional decline at 1st visit**, n = 131				*p* = 0.194
Slow progressors (ΔFS < 0.5)	192.48 ± 156.69	134.00	91.00–220.00	
Fast/average progressors (ΔFS ≥ 0.5),	163.70 ± 141.18	111.00	69.00–214.00	

Results were presented as mean ± SD, medians, and quartiles (Q1–Q3) for serum CK level in males; significance was calculated with Mann–Whitney tests. Bold values show *p*-values < 0.05. **Legend**: **CK**—creatine kinase; **ALS**—amyotrophic lateral sclerosis; **SD**—standard deviation; **UL**—upper limb; **LL**—lower limb; **EMG**—electromyography.

**Table A5:** Demographics and ALS clinical variables in association with 90th percentile (P90) CK for men (736 U/L).

Variables	Total (*n* = 189)	CK < P90 (*n* = 170)	CK ≥ P90 (*n* = 19)	*p*-Value
**Age at diagnosis (years)**, n = 189				***p* = 0.013** (a)
Mean ± SD	59.62 ± 13.04	60.41 ± 12.53	52.63 ± 15.63	
Median	60.00	61.00	54.00	
Q1–Q3	50.00–71.00	51.00–71.00	42.00–66.00	
**Onset site** n (%), n = 189				*p* > 0.990 (b)
Spinal	153 (81.0%)	137 (80.6%)	16 (84.2%)	
Bulbar	36 (19.0%)	33 (19.4%)	3 (15.8%)	
**Fasciculations at onset** n (%), n = 176	81 (46.0%)	73 (46.2%)	8 (44.4%)	*p* > 0.990 (b)
**Needle EMG** n (%) n = 168				*p* > 0.990 (b)
UL or LL abnormal	25 (14.9%)	23 (15.3%)	2 (11.1%)	
UL and LL abnormal	143 (85.1%)	127 (84.7%)	16 (88.9%)	
**Disease duration** (months), n = 189				*p* = 0.090 (c)
Mean ± SD	17.72 ± 20.50	16.62 ± 17.00	27.59 ± 39.53	
Median	11.53	11.29	14.26	
Q1–Q3	6.57–20.01	6.37–19.48	9.89–24.80	
**Functional decline rate at 1st visit**, n = 189				*p* = 0.060 (c)
Mean ± SD	0.79 ± 0.89	0.83 ± 0.92	0.43 ± 0.37	
Median	0.48	0.51	0.34	
Q1–Q3	0.24–0.97	0.25–1.01	0.17–0.52	

Results were presented as n (%) for categorical variables and Min–Max, mean ± SD, medians, and quartiles (Q1–Q3) for continuous variables; significance was calculated with (a) *t*-test, (b) Fisher exact test, and (c) Mann–Whitney tests. Bold values show *p*-values < 0.05. **Legend**: **CK**—creatine kinase; **ALS**—amyotrophic lateral sclerosis; **SD**—standard deviation; **UL**—upper limb; **LL**—lower limb; **EMG**—electromyography.

**Table A6:** Demographics and ALS clinical variables in association with 90th percentile (P90) CK for females (399 U/L).

Variables	Total (*n* = 131)	CK < P90 (*n* = 118)	CK ≥ P90 (*n* = 13)	*p*-Value
**Age at diagnosis (years)**, n = 131				*p* = 0.682 (a)
Mean ± SD	63.87 ± 12.53	63.72 ± 12.84	65.23 ± 9.62	
Median	66.00	65.00	68.00	
Q1–Q3	55.00–75.00	55.00–73.00	57.00–72.00	
**Onset site** n (%), n = 131				***p* = 0.004** (b)
Spinal	86 (65.6%)	73 (61.9%)	13 (100%)	
Bulbar	45 (34.4%)	45 (38.1%)	0 (0%)	
**Fasciculations at onset** n (%), n = 123	29 (23.6%)	26 (23.6%)	3 (23.1%)	*p* > 0.990 (b)
**Needle EMG** n (%) n = 116				*p* = 0.637 (b)
UL or LL abnormal	14 (12.1%)	12 (11.5%)	2 (16.7%)	
UL and LL abnormal	102 (87.9%)	92 (88.5%)	10 (83.3%)	
**Disease duration** (months), n = 131				*p* = 0.530 (c)
Mean ± SD	15.19 ± 13.10	15.07 ± 13.31	16.24 ± 11.45	
Median	11.56	11.06	11.96	
Q1–Q3	6.97–19.06	6.97–16.92	9.66–19.65	
**Functional decline rate at 1st visit**, n = 131				*p* = 0.712 (c)
Mean ± SD	0.94 ± 1.24	0.95 ± 1.28	0.82 ± 0.71	
Median	0.64	0.65	0.60	
Q1–Q3	0.38–1.14	0.39–1.14	0.26–1.00	

Results were presented as n (%) for categorical variables and Min–Max, mean ± SD, medians, and quartiles (Q1–Q3) for continuous variables; significance was calculated with (a) *t*-test, (b) Fisher exact test, and (c) Mann–Whitney tests. Bold values show *p*-values < 0.05. **Legend**: **CK**—creatine kinase; **ALS**—amyotrophic lateral sclerosis; **SD**—standard deviation; **UL**—upper limb; **LL**—lower limb; **EMG**—electromyography.

**Table A7:** Descriptive of the respiratory function variables at baseline in association with 90th percentile (P90) adjusted to sex (P90 males = 736 U/L; P90 females = 399 U/L).

Characteristics	Total	CK < P90 (*n* = 288)	CK ≥ P90 (*n* = 32)	*p*-Value
**Forced vital capacity (FVC%)**, n (%), n = 181				***p* = 0.038** (a)
Mean ± SD	82.83 ± 23.72	81.65 ± 23.55	94.18 ± 23.03	
Median	86.00	85.15	100.00	
Q1–Q3	68.00–98.34	68.00–97.00	77.70–112.00	
**Phrenic CMAP amplitude (mV)**, n (%), n = 209				*p* = 0.440 (c)
Mean ± SD	0.52 ± 0.28	0.52 ± 0.28	0.48 ± 0.29	
Median	0.50	0.51	0.44	
Q1–Q3	0.32–0.66	0.32–0.66	0.25–0.69	
**ALSFRS-R respiratory sub-scores**, n (%), n = 320				*p* = 0.470 (d)
Q10 + Q11 + Q12 = 12	237 (74.1%)	215 (74.7%)	22 (68.8%)	
Q10 + Q11 + Q12 < 12	83 (25.9%)	73 (25.3%)	10 (31.3%)	

Results were presented as n (%) for categorical variables and Min–Max, mean ± SD, medians, and quartiles (Q1–Q3) for continuous variables; significance was calculated with (a) *t*-tests, (c) Mann–Whitney tests, and (d) chi-square tests. Bold values show *p*-values < 0.05. **Legend**: **CK**—creatine kinase.
